# Composite Sorbents Based on Chitosan Polymer Matrix and Derivatives of 4-Amino-N′-hydroxy-1,2,5-oxadiazole-3-carboximidamide for Uranium Removal from Liquid Mineralized Media

**DOI:** 10.3390/gels11010024

**Published:** 2025-01-01

**Authors:** Anna I. Matskevich, Konstantin V. Maslov, Veronika A. Prokudina, Daria D. Churakova, Oleg Yu. Slabko, Dmitry K. Patrushev, Nikita S. Markin, Eduard A. Tokar’

**Affiliations:** 1 Institute of Natural Sciences and Technosphere Safety, Sakhalin State University, Sakhalin Region, 693000 Yuzhno-Sakhalinsk, Sakhalin Oblast, Russia; mysmatskevich@mail.ru (A.I.M.); maslov.kv@dvfu.ru (K.V.M.); veronikaprokudina2002@mail.ru (V.A.P.); patrushev.dk@dvfu.ru (D.K.P.); markkin.ns@gmail.com (N.S.M.); 2 Institute of High Technologies and Advanced Materials, Far Eastern Federal University, 690922 Vladivostok, Primorsky Krai, Russia; dawa.cyratava@mail.ru (D.D.C.); slabko.oyu@dvfu.ru (O.Y.S.)

**Keywords:** chitosan, sorption, amidoxime, uranium, 2,5-oxadiazoles, polymers

## Abstract

Composite adsorbents based on a natural biopolymer matrix of chitosan, to which 4-amino-N′-hydroxy-1,2,5-oxadiazole-3-carboximidamide and its Se derivative were attached, were synthesized. A complex of physicochemical analysis methods indicates that the direct introduction of a matrix with high ionic permeability into the reaction mixture contributes to the formation of homogeneous particles of composite with developed surface morphology, which enhances the kinetic and capacitive parameters of uranium sorption in liquid media. It has been established that the direct introduction of a matrix with high ionic permeability into the reaction mixture contributes to the formation of homogeneous particles with a developed surface morphology, which enhances the kinetic and capacitive parameters of uranium sorption in liquid media. The synthesized materials had increased sorption-selective properties towards uranium in the pH range from 4 to 9 under static sorption conditions. The formation of the Se derivative of amidoxime during its attachment to the polymer matrix (Se-chit) contributes to the creation of a more chemically stable and highly effective adsorbent, compared to the direct binding of 4-amino-N′-hydroxy-1,2,5-oxadiazole-3-carboximidamide with chitosan (43AF-chit). The optimal parameters for the synthesis of materials were established. It was demonstrated that the ratio of amidoxime to chitosan should be within the range of 2:1 to 1:2. As the mass content of chitosan increases, the material gradually dissolves and transforms into a gel, resulting in the formation of liquid radioactive waste with a complex chemical composition. It was found that the kinetic sorption parameters of composite materials increase 2–10 times compared to those of non-composite materials. The sorption capacity of uranium in solutions with pH 6 and pH 8 can reach approximately 400–450 mg g^−1^. Under dynamic sorption conditions, the effective filtration cycle values (before uranium slips into the filtrate ≥ 50%) improve significantly when transitioning from a non-composite adsorbent to a composite one: increasing from 50 to 800 b.v. for pH 6 and from 2700 to 4000 b.v. for pH 8. These results indicate that the synthesized sorbents are promising materials for uranium removal from liquid media, suitable for both purification and the recovery of radionuclides as valuable raw materials.

## 1. Introduction

Removing uranium from liquid media has become an increasingly relevant problem due to the need for sustainable energy sources and, as a consequence, the growing interest in nuclear energy. Uranium is a primary component of fuel for nuclear reactors, and its selective extraction from various sources—such as groundwater, wastewater, and ore solutions—for both purification and recovery as a valuable raw material, is becoming a key area of research [1,2].

Among the various methods for removing uranium from liquid media, several techniques stand out: extraction [3,4], electrocoagulation [5], chemical precipitation [6], membrane filtration [7], photocatalytic reduction [8], sorption [9,10,11], etc. Although a wide range of approaches is available, many come with significant limitations that impede their practical application in real conditions. For example, the extraction method that involves the use of organic solvents (tributyl phosphate) [12] is a traditional technique for uranium removal and is widely utilized in industry. Despite its high efficiency, this process requires complex cleaning procedures and methods for handling volatile organic compounds, which result in stricter safety and environmental protection requirements. Additionally, extraction methods, similar to sedimentation methods, generate secondary liquid radioactive waste [6] with a complex chemical composition.

The advantages of membrane technology include low operating costs and the potential for membrane recycling. However, the high cost of membranes and the necessity for regular maintenance are significant drawbacks to this approach. Additionally, most methods are characterized by a significant decrease in the selectivity of uranium removal from solutions with high mineralization, leading to the formation of a wide range of complex ionic forms of uranium [13].

Promising results have been achieved using the adsorption method for uranium removal [14], which is characterized by its simplicity, ecological safety, and low cost. The advantages of the method are provided by the use of highly selective natural and synthetic adsorbents towards uranium, the selection of which is based on the chemical composition of the systems being cleaned and the conditions of the separation or purification processes. Layered aluminosilicates, such as montmorillonite [15] and kaolinite [16], can be used as natural adsorbents. Their advantages include high mechanical strength, absence of negative effects on the environment, and low cost. However, they are characterized by low selectivity for uranium in multicomponent solutions with high mineralization, as evidenced by their capability to sorb various elements (heavy metals and radionuclides) [17].

Synthetic materials exhibit enhanced sorption-selective characteristics compared to natural sorbents. This class of materials can be roughly categorized into organic, inorganic, and composite types. This includes organometallic frameworks [18], oxides and hydroxides of transition metals (such as Mn [19], Fe [20], Ti [21,22], Al [23], etc.), polymers [24], carbon adsorbents [25], magnetic materials [26], biomatrix [27], and others.

In our opinion, synthetic adsorbents with chelating functional groups such as amino [28], sulfhydryl, carboxyl [29], amidoxime, and imidiacetate are particularly noteworthy. Their enhanced sorption-selectivity enables them to effectively bind uranium and its complex ions through ion exchange and complexation reactions [20,21,22]. To develop materials with enhanced sorption capacity (provided by a large number of sorption centers) and mechanical strength, composite sorbents are often synthesized. These sorbents are typically produced through the amidoximation of various types of matrices: polyethylene [30,31], carbon nanotubes [32], chitosan [33], cellulose [34], and others [35]. This approach enables the introduction of specific sorption-selective properties to existing matrices that previously did not possess them.

The authors of [28] showed that grafting of chitosan and acrylonitrile to graphite leads to the formation of a large number of sorption centers, which can subsequently be additionally functionalized by OH-N=C<NH_2_ groups through an amidoximation reaction. The authors demonstrated that the material obtained through this method exhibits enhanced sorption properties for uranium.

The directed synthesis of the composite enabled the selective extraction of up to 200 mg g^−1^ of uranium from liquid media with a pH of 5–7, with adjustable selectivity based on the presence of competing ions. To prevent the dispersion and degradation of sorbents based on polymer matrices under dynamic sorption conditions, nanoscale magnetic particles were synthesized through the co-deposition of magnetite and chitosan. These particles were then subjected to additional crosslinking with epichlorohydrin and grafted with diethylenediamine fragments, which introduced additional functional groups (–NH_2_ and –OH) [36,37]. This approach enabled a tenfold increase in the adsorption capacity of the adsorbents toward uranium (up to 150 mg g^−1^) compared to both pure magnetite/magnetic chitosan [38,39] and chitosan with enhanced crosslinking of the polymer network [40].

In [41], the authors conducted the functionalization of the chitosan matrix through both the direct amidoximation reaction of hydroxyl groups and the grafting of additional organic radicals (polyethylene polyamine/polydopamine). This enabled the formation of a significant number of adsorption centers (SOEU—up to 470 mg g^−1^).

Thus, sorption materials based on amidoxime compounds, and their composites are being actively researched for practical applications, demonstrating high effectiveness in removing uranium from various types of liquid media. However, in the literature, many developed adsorbents are described in a limited manner, which impedes a full assessment of their performance in real conditions for radionuclide removal from complex chemical media. Simultaneously, an important scientific and technical problem lies in reducing the cost of the initial components used in adsorbent production and simplifying the synthesis method itself.

The aim of this work is to develop any approaches for the synthesis of new sorption materials based on a polymer matrix (chitosan) and derivatives of 4-amino-N′-hydroxy-1,2,5-oxadiazole-3-carboximidamide. Previous studies have demonstrated that a one-stage polycondensation reaction between SiO_2_ and 4-amino-N′-hydroxy-1,2,5-oxadiazole-3-carboximidamide leads to the formation of a polymer material with increased chemical resistance, attributed to the presence of strong diselenide bridges. This material exhibits enhanced sorption-selective properties toward uranium when compared to most known adsorbents [11].

Grafting of an organic radical onto a polybase will significantly enhance the kinetic parameters of the radionuclide sorption, owing to the high ionic permeability of the matrix and the development of a well-structured chitosan surface morphology. Additionally, the grafting approach, which involves attaching the sorption-active component to chitosan and simultaneously precipitating it, will produce mechanically robust granules with uniformly distributed sorption centers throughout the entire volume of the adsorbent. This approach will also enhance the sorption capacity and improve the overall performance of the adsorbents, making them suitable for use in dynamic uranium sorption conditions.

## 2. Results and Discussion

At the first stage of the research, the optimal conditions for the synthesis of adsorbents with increased sorption-selective properties toward uranium were determined. Two approaches to the synthesis of the materials were employed for this purpose. In the first approach, the polycondensation reaction of the Se derivative of 4-amino-N′-hydroxy-1,2,5-oxadiazole-3-carboximidamide was conducted in the presence of a polymer matrix (chitosan), which enhances ionic permeability and increases the contact surface area. This method of adsorbent preparation has already demonstrated its effectiveness toward uranium sorption [11]. In the second approach, to simplify the synthesis process, the composites were obtained by directly binding an organic radical (4-amino-N′-hydroxy-1,2,5-oxadiazole-3-carboximidamide), which contained the primary sorption groups (–NH_2_ and –OH), to an organic chitosan matrix.

To assess how synthesis conditions affect the sorption-selective characteristics of materials towards uranium, we created a series of adsorbents with varying ratios of the initial components. Additionally, the effects of the curing temperature on the materials’ properties were analyzed (Table 1), as this can significantly impact the crosslinking of the polymer network and, consequently, the sorption characteristics of the materials. The previously described adsorbent Se-init [11], which demonstrated improved sorption-selective properties towards uranium, was used as a comparison sample.

### 2.1. Physicochemical Characteristics of the Sorbent

**Figure 1:**
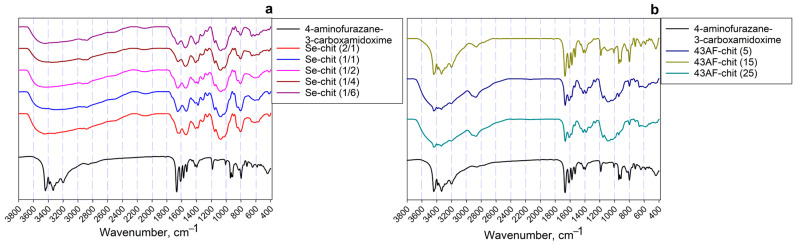
IR-spectra of synthesized materials: (**a**) based on Se derivative of N′-hydroxy-1,2,5-oxadiazole-3-carboximidamide (Se-chit (x/y)), (**b**) based on N′-hydroxy-1,2,5-oxadiazole-3-carboximidamide (43AF–chit(x)).

The infrared spectra (IR-spectra) of the synthesized composite materials and the initial 4-amino-N′-hydroxy-1,2,5-oxadiazole-3-carboximidamide are presented in Figure 1. Independently of the method for composite preparation, all characteristic absorption bands corresponding to chitosan and 4-amino-N′-hydroxy-1,2,5-oxadiazole-3-carboximidamide are observed in each spectrum. This indicates the uniform formation of the sorption-active component on the surface of the polymer matrix through covalent binding, ensuring the preservation of functional amidoxime radicals.

In the region of 3000–3600 cm^−1^, a wide peak is observed, corresponding to both asymmetric and symmetric valence vibrations of hydrogen bonds present in the composition of chitosan and amidoxime. These vibrations include: –O–H (3440 cm^−1^), –NH_2_ (3200 cm^−1^), and the interactions O–H***N and N–H***O. At the same time, the absorption bands of the amidoxime groups in the Se-chit (x/x) samples (Figure 1a) completely overlap. This phenomenon is probably due to a deeper and stronger interaction between the initial components of the mixture compared to the 43AF-chit (x) samples, where the formation of amidoxime film occurs to a greater extent on the matrix surface. In the region of 2900 cm^−1^ and 950–1100 cm^−1^, wide bands are observed, corresponding to vibrations in the C–H and C–O bonds of chitosan. The intensity of these band increases as the mass content of the matrix in the composite increases (Figure 1b). The absorption bands at 1668 cm^−1^ and 1357 cm^−1^, which are characteristic of each type of composite, correspond to the valence vibrations of the C=N bonds in the carboxamide group and C–N bonds included in the amidoxime radical. The absorption bands in the regions of 1616 cm^−1^ and 1575 cm^−1^ correspond to the valence vibrations of the C=N bond in the furazan cycle. A distinctive feature of Se-containing composites is the presence of absorption bands in the 412 cm^−1^ and 489 cm^−1^ regions associated with vibrations of the Se-Se and N-Se bands, respectively. Additionally, an important feature is the presence of an absorption band at 1548 cm^−1^, which corresponds to vibration of the Se=C–N.

**Figure 2:**
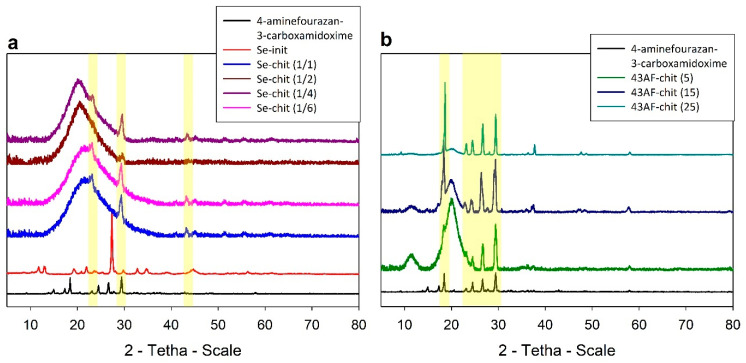
Diffractograms of synthesized materials: (**a**) based on Se derivative of N′-hydroxy-1,2,5-oxadiazole-3-carboximidamide (Se-chit (x/y)), (**b**) based on N′-hydroxy-1,2,5-oxadiazole-3-carboximidamide (43AF–chit(x)).

The X-ray diffractograms of the obtained composites (Figure 2) show a clearly pronounced halo in the range of 20–23 Å, corresponding to the amorphous phase of the polymer matrix. The intensity of this halo increases proportionally with the mass fraction of chitosan in the sorbent: Se-chit (1/1) < Se-chit (1/2) < Se-chit (1/4) < Se-chit (1/6) and 43AF-chit (25) < 43AF-chit (15) < 43AF-chit (5)

The X-ray diffractograms of the obtained composites (Figure 2) show a clearly pronounced amorphous halo (15 < 2θ < 28), corresponding to the amorphous phase of the polymer matrix. The intensity of this halo increases proportionally with the mass fraction of chitosan in the sorbent: Se-chit (1/1) < Se-chit (1/2) < Se-chit (1/4) < Se-chit (1/6) and 43AF-chit (25) < 43AF-chit (15) < 43AF-chit (5) (Table 1).

The decrease in the intensity of the reflex corresponding to the Se-init phase (Figure 2a), in contrast to the crystalline phase of N′-hydroxy-1,2,5-oxadiazole-3-carboximidamide observed in the 43AF-chit(x) samples (Figure 2b), is probably attributable to the peculiarities of forming and binding the sorption-active component during the synthesis of the materials. In the production of Se-chit (x/y) composites, all components of the mixture were brought to a homogeneous state, ensuring the formation of particles with uniform chemical composition throughout the volume.

Unlike the composites of a 43AF-chit(x) series, where the amidoxime coating was formed on the surface of the chitosan, probably through covalent binding. The film thickness increased as the mass content of amidoxime increased. This observation is supported by the increase in the intensity of the corresponding reflex at 2θ = 19 deg.

**Figure 3:**
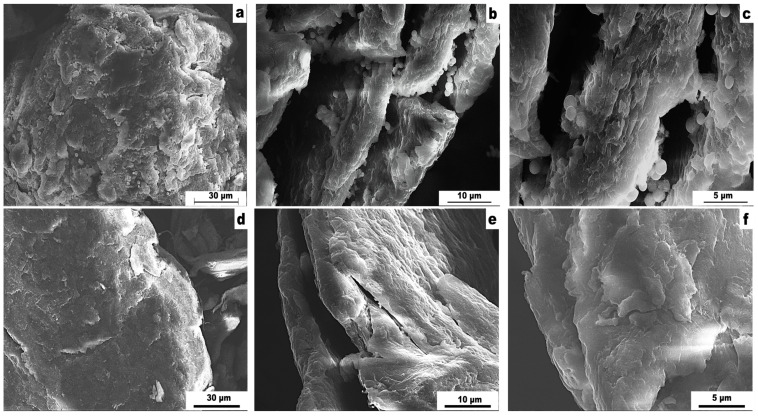
SEM-images of synthesized materials: (**a**–**c**) based on Se derivative of N′-hydroxy-1,2,5-oxadiazole-3-carboximidamide (Se-chit (x/y)), (**d**–**f**) based on N′-hydroxy-1,2,5-oxadiazole-3-carboximidamide (43AF–chit (x)).

The scanning electron microscopy (SEM) images of the Se-chit (1/1) and 43-AF-chit (25%) composites are presented in Figure 3. The surface morphology for the other adsorbents is similar to that of the presented samples. The morphology characterizes an irregular structure with the inclusion of particles with irregular floccular shapes (Figure 3d–f) and spherogranulated shapes (Figure 3a–c). In both types of composites, the formation of macropores is observed, which are visible in the micrographs. The presence of such pores can enhance the number of sorption-active centers.

**Figure 4:**
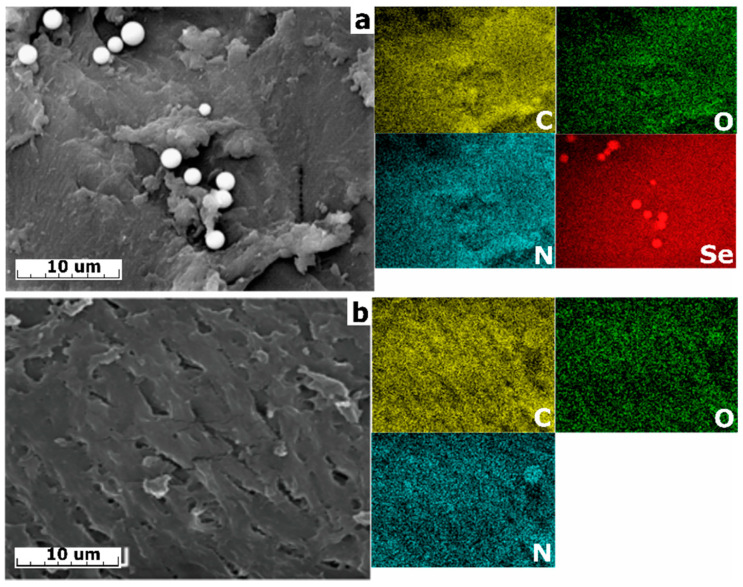
The results of EDX analysis of composite materials: (**a**) Se-chit (1/1), (**b**) 43-AF-chit (25%).

The energy dispersive spectroscopy (EDX) images presented in Figure 4 show a uniform distribution of all components in the mixture, indicating the homogeneity of the composite materials’ formation process. In the case of Se-chit (x/y), the spherogranulated agglomerations appears as large particles of Se derived from amidoxime (Se-init) forming on the surface of the composite. This phenomenon is previously undescribed in the literature.

**Figure 5:**
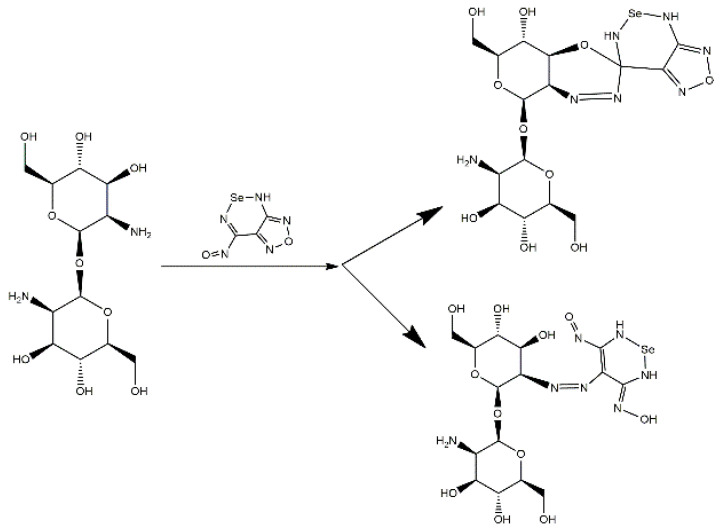
The scheme of formation of composites based on 4-amino-N′-hydroxy-1,2,5-oxadiazole-3-carboximidamide (43AF-chit (x)).

**Figure 6:**
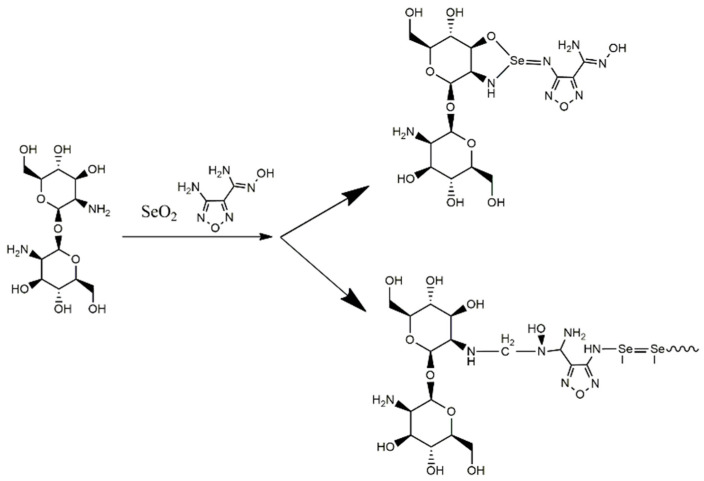
The scheme of formation of composites based on Se derivative of 4-amino-N′-hydroxy-1,2,5-oxadiazole-3-carboximidamide (Se-chit (1/1)).

Based on the results of the investigation of the physicochemical properties of the materials, an assumption was made regarding the reaction scheme and the structure of the elementary polymer units of the synthesized materials (Figure 5 and Figure 6). However, to establish a more accurate molecular structure, a broader range of physicochemical studies is required, which is beyond the scope of this work and will be describe in our future studies.

The use of chitosan as a matrix for composites is caused by two key factors. Firstly, chitosan increases the mechanical strength of the materials. Secondly, it is characterized by high kinetic sorption parameters, which are provided by the high ionic permeability of the polymer. The properties of chitosan can be further enhanced by crosslinking to the polymer network. This can be achieved by binding free amino groups in the chitosan chains with crosslinking agents, such as dicarboxylic acids, as well as through thermal treatment, which promotes the removal of bound and intercrystalline water, along with other volatile reaction products.

**Figure 7:**
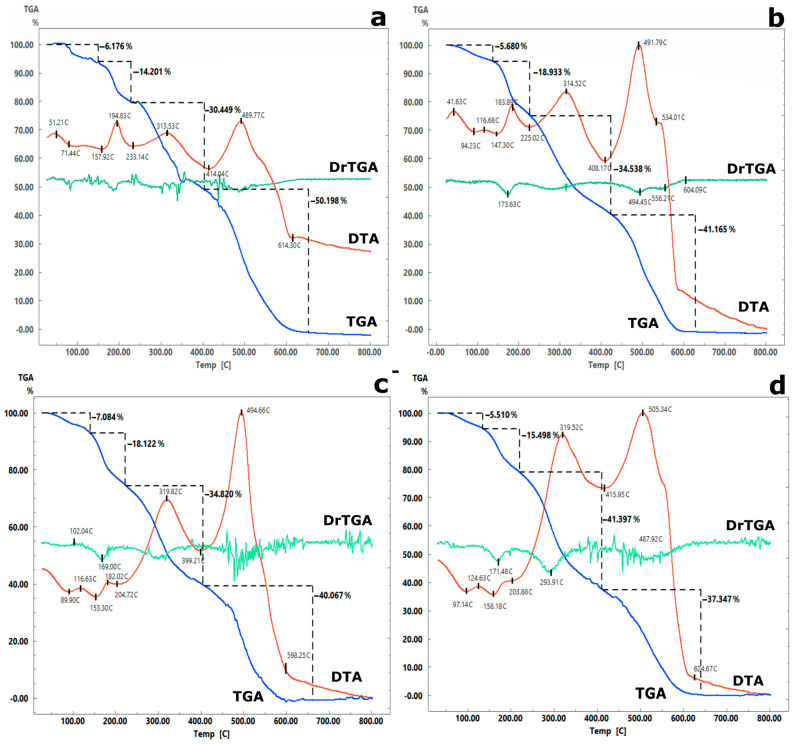
Thermograms of composite materials of the series: (**a**) Se-chit (1/1), (**b**) Se-chit (1/2), (**c**) Se-chit (1/4), (**d**) Se-chit (1/6).

**Figure 8:**
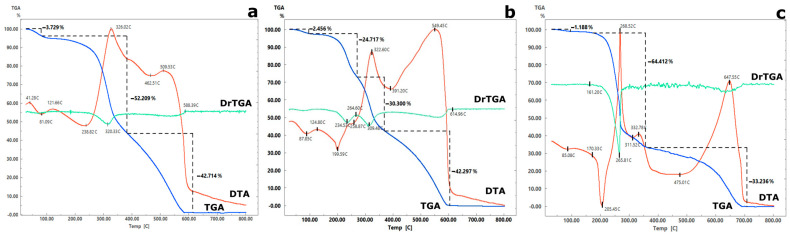
Thermograms of composite materials of the series: (**a**) 43AF-chit (5), (**b**) 43AF-chit (15), (**c**) 43AF-chit (25).

The results of the research [42] indicate that chitosan films heated after their conversion to the polybasic form exhibit lower swelling and greater strength compared to the original films. At the same time, the strength of heated chitosan film was comparable to the strength of chitosan modified with crosslinking agents. Additionally, the permeability of the films was maintained, ensuring that the active component remained accessible to the solution. Considering this, to obtain adsorbents with increased mechanical strength and sorption-selective properties, the synthesized materials were subjected to additional temperature treatment (Table 1). The temperature treatment conditions were selected based on the thermogravimetric analysis of the unheated samples (Figure 7 and Figure 8).

Regardless of the ratio of the initial components (Figure 7 and Figure 8), the thermograms show a wide area of endothermic effects in the temperature range of 40–200 °C, mainly associated with the removal of weakly bound (105–130 °C) and intracrystalline (130–200 °C) water. At the same time, the weight loss for the 43AF-chit (x) composites did not exceed 5%, while the series of Se-chit (x/y) materials exhibited weight loss amounts of 15–20%. An increase in temperature to 200 °C initiated thermal oxidative degradation, leading to the decomposition of the organic component and resulting in the greatest mass loss of the sorbent. Based on the results obtained, the temperature regime of curing composites should not exceed 200 °C.

### 2.2. Sorption Selective Properties

To establish the optimal parameters for the synthesis of composites, we investigated the dependence of the sorption-selective properties of the adsorbents of uranium on their physicochemical properties.

**Figure 9:**
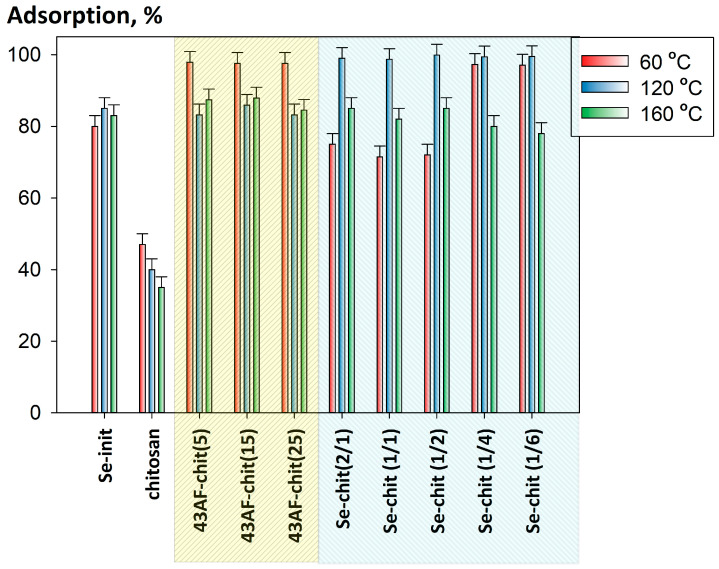
Relationship between the efficiency of uranium removal from 0.1 M NaNO_3_ solutions at pH 6 and the curing temperature of the composites (V:m = 1000 mL g^−1^).

Figure 9 presents the relationship between the efficiency of uranium removal from 0.1 M NaNO_3_ solutions at pH 6 and the curing temperature of the composites. The initial chitosan and Se-init, heated at the appropriate temperatures, were used as comparison samples. The results show that the highest values of uranium adsorption for the adsorbents in the 43AF-chit(x) series are achieved with a curing temperature of 60 °C. This is probably attributable to the mass content of polybasic, which contains a significant number of adsorption centers (–NH_2_ and –OH), which are capable of binding a complex of uranium ions. This is indirectly confirmed by the results of uranium adsorption evaluated for the Se-chit (1/4) and Se-chit (1/6) samples, which were also cured at 60 °C.

The decrease in adsorption efficiency for the 43AF-chit(x) samples cured above 60 °C is probably due to the uncontrolled crosslinking process of the chitosan polymer network, which leads to a reduction in the number of sorption centers. For the Se-chit (x/y) samples, which contain significantly less chitosan, the best sorption values are achieved at a curing temperature of 120 °C. This is probably also related to the specific nature of the thermopolymerization process of the sorption-active components, namely amidoxime and chitosan.

**Figure 10:**
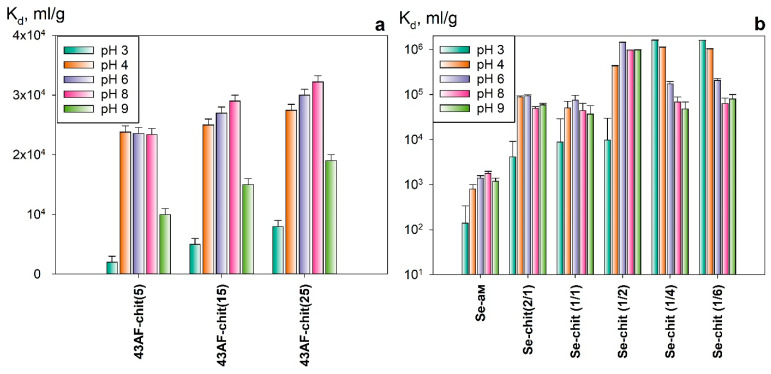
Distribution coefficients for uranium across different ratios of sorption-active components and matrix at varying pH levels: (**a**) adsorbents from the 43AF-chit (x) series; (**b**) adsorbents from the Se-chit (x/y) series, cured at 60 °C and 120 °C, respectively (ratio V:m = 1000 mL g^−1^).

Figure 10 presents diagrams illustrating the relationship between the distribution coefficients of uranium for the removal of the radionuclide from solutions with a pH 3–9, using 43AF-chit (x) and Se-chit (x/y), which have been cured at 60 and 120 °C, respectively. By using 4-amino-N′-hydroxy-1,2,5-oxadiazole-3-carboximidamide as a sorption-active component, the values of the uranium distribution coefficient (*K_d_*) increase with the content of amidoxime in the composite. The highest values of sorption efficiency were achieved in the pH range 4–8, exceeding 98%. This increase is primarily attributed to the amidoxime component and is consistent with previous research for the Se derivative of N′-hydroxy-1,2,5-oxadiazole-3-carboximidamide (Se-init) [11].

Adsorbents based on the Se derivative of 4-amino-N′-hydroxy-1,2,5-oxadiazole-3-carboximidamide (Figure 10b) are characterized by an increased affinity for uranium across the entire pH range of solutions that were studied. The *K_d_* value of uranium is approximately 10^5^–10^6^ mL g^−1^, attributed to the increased availability of adsorption centers due to the developed surface morphology (Figure 3a–c) and the high ionic permeability of the matrix. Increasing the chitosan content in the composite (Se-chit (1/2) < Se-chit (1/6)) results in a shift toward highly selective sorption at pH < 7. It is probably due to the characteristics of the polymer matrix. However, significant dissolution of the composites and their transition into a gel-like form was observed after sorption. A similar effect was observed for the 43AF-chit (x) samples. It limits the possibility of a practical use for the materials described because the dissolution may result in the formation of secondary liquid radioactive waste with a complex chemical composition. The highest uranium sorption values in the pH range of 4–9, along with improved chemical and mechanical stability, were achieved with the samples Se-chit (2/1), Se-chit (1/1), and Se-chit (1/2), which were cured at 120 °C. This enhancement is probably due to increased crosslinking of the polymer network resulting from the removal of intracrystalline water molecules and the formation of additional methylene bridges (Figure 8). A decrease in uranium sorption efficiency to 70–80% on samples cured at 160 °C is probably due to the onset of the process of thermal oxidative degradation and a reduction in the number of sorption centers.

Based on the presented results, further experiments were conducted using Se-chit (2/1), Se-chit (1/1), and Se-chit (1/2), cured at 120 °C. Se-init was used as a comparison sample.

One of the key problems that this work aimed to solve is the removal of uranium from solutions with high salinity and complex chemical compositions. In such systems, uranium exists as a wide variety of complex ions, which is attributed to the radionuclide’s tendency to form mono- and multinuclear complexes with various anions [43].

**Figure 11:**
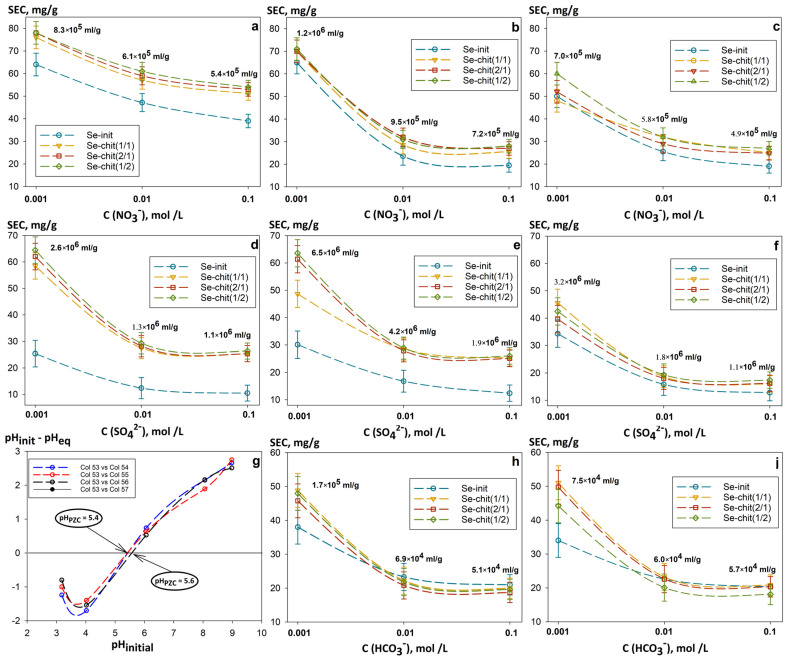
Dependence of the static exchange capacity of uranium on the type and concentration of competing anions in solutions: (**a**,**d**) pH 4, (**b**,**e**,**h**) pH 6, (**c**,**f**,**i**) pH 8; (**g**) the value of the zero charge point of sorbents (pH_pzc_), (V:m = 1000 mL g^−1^).

Figure 11 illustrates the dependence of the static exchange capacity (*SEC*) value of uranium on the type and concentration of the most common competing anions, obtained in model solutions at pH 4 (Figure 11a,d), pH 6 (Figure 11b,e,h), and pH 8 (Figure 11c,f,j). The ions SO_4_^2−^ and HCO_3_^−^ were chosen as the competing anions with the greatest influence, while NO_3_^−^ was used as the reference anion, because it has the least detrimental effect on uranium sorption. There is a general tendency for the adsorption of uranium to decrease with an increase in the concentration of anions up to 0.1 mol L^−1^, probably due to the competing ion exchange reactions of the anions themselves. This effect is slightly enhanced by increasing the pH of the solution from 4 to 8, which is attributed to the negatively charged surface of the composites, with *pH_pzc_* values varying from 5.4 to 5.6 (Figure 11g). Despite this, the order of *SEC* magnitude at a single pH level remains practically unchanged: 10^5^–10^6^ mL g^−1^ for SO_4_^2−^ and 10^4^ mL g^−1^ for HCO_3_^−^. The competing effects of the anions are accompanied by a decrease in the SEC value of uranium in the order NO_3_^−^ ≥ SO_4_^2−^ > HCO_3_^−^.

The main influence of hydrocarbonates occurs during the formation of multinuclear complexes such as [UO_2_(CO_3_)_3_]^4−^, [UO_2_(CO_3_)_4_]^3−^, [UO_2_(CO_3_)_2_(H_2_O)_2_]^2−^ [29]. Such complexes, from the perspective of electrolytic interactions, are expected to selectively bind to the positively charged surfaces of adsorbents. Probably, this effect is offset by the high ionic charge, which requires a larger number of adsorption centers. In solutions at pH 6 and pH 8, a significant negative influence can be observed with positively charged uranium hydroxocomplexes of the types [(UO_2_)_3_(OH)_5_]^+^, [(UO_2_)_4_(OH)_7_]^+^, and [(UO_2_)_3_(OH)_2_]^4+^ [44]. However, such an effect was not observed, which was probably due to the binding of these ions during the deprotonation of –NH_2_ and –OH groups that are part of both the sorption-active component and the chitosan matrix

**Figure 12:**
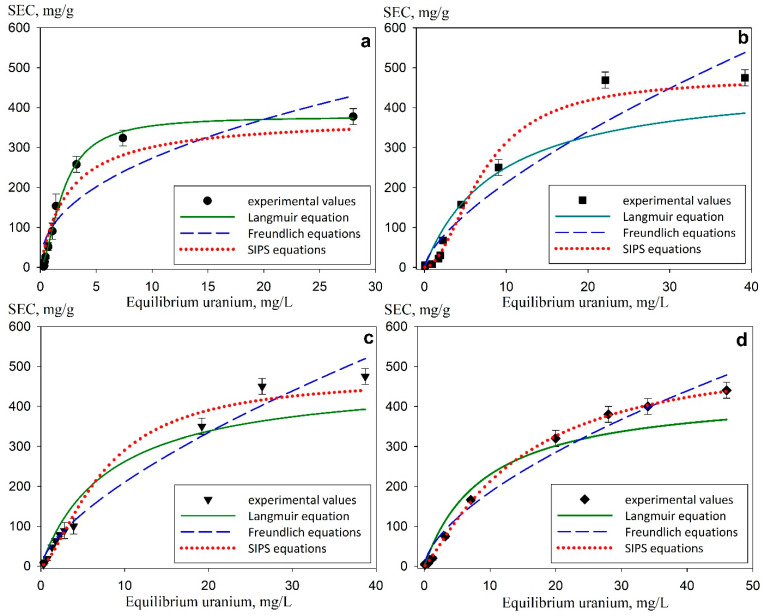
Isotherms of uranium sorption from model solutions with pH 6 and approximation of the experimental values by the Langmuir, Freundlich, and SIPS equations: (**a**) Se-init, (**b**) Se-Hit (2/1), (**c**) Se-Hit (1/1), (**d**) Se-Hit (1/2).

Figure 12 presents the adsorption isotherms obtained in the model 0.01 M NaNO_3_ solutions with pH 6 for Se-init, Se-Hit (2/1), Se-Hit (1/1), Se-Hit (1/2) sorbents. The graphs also show the curves of approximation of experimental values by the Langmuir, Freundlich and SIPS equations. Table 2 shows the corresponding coefficients. The isotherms can be attributed to the L-type [45], thus indicating high affinity of the adsorbents to uranium, including the region of its low concentration.

Table 2 shows the corresponding coefficients of the Freundlich, Langmuir, and SIPS equations obtained by nonlinear regression of experimental values of uranium sorption. According to the data presented, the values of *K*_f_ and *m* for composite materials remain practically constant regardless of the initial components of the sorbents’ ratio. This indicates the homogeneity of the sorbent sorption centers located both on the surface and in the grain volume. The experimental values obtained for composites, according to the correlation coefficient (>0.98), are best approximated by the SIPS equation, unlike the non-composite Se-init. This indicates that the saturation of the monolayer is significantly influenced by the heterogeneity of the adsorption centers. A significant difference is observed in the maximum sorption capacity values (*G*_max_), the highest values of which were obtained for composite adsorbents. This fact is attributed to the greater physicochemical availability of sorption centers. There is a decrease in the values of *K*_l_ and *K*_lf_ in the series Se-Hit (1/1) > Se-Hit (1/2) > Se-Hit (2/1), which may indicate a decrease in the mechanical strength and destruction of the sorbents caused by peptization of the polybase.

**Figure 13:**
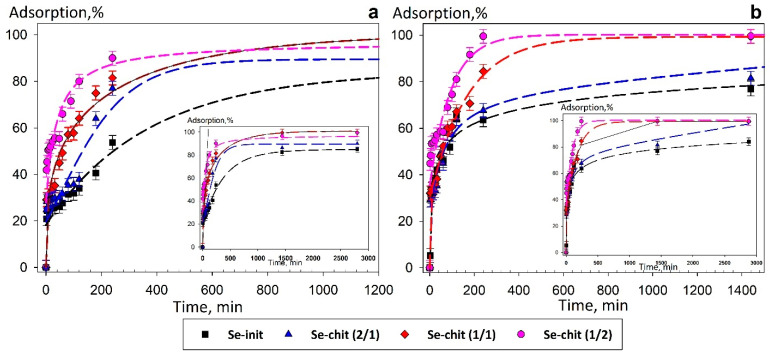
Dependence of the efficiency of uranium extraction on the type of sorbent for model solutions with: (**a**) pH 6, (**b**) pH 8.

Figure 13 and Table 3 present the results of the evaluation of the kinetic parameters of uranium sorption from solutions with pH 6 (Figure 13a) and pH 8 (Figure 13b) for composite materials and for Se-init. The kinetic sorption curves indicate that the presence of a highly ion-permeable matrix contributes to an increase in the kinetic parameters of sorption. There is a significant decrease in the half-exchange time (T_1/2_), as well as an increase in the values of the diffusion coefficient (*D_i_*) and *K_d_* (Table 3) in the row Se-init → Se-chit (2/1) → Se-chit (1/1) → Se-chit (1/2). This is due to the participation of additional sorption centers –NH_2_ and –OH of the chitosan polymer matrix, capable of dehydration followed by capture of uranium ion complexes. Using Se-chit (1/1), Se-chit (1/2), adsorption equilibrium can be achieved within 3–4 h, which was provided by an optimal ratio of the sorption-active component and the matrix.

**Figure 14:**
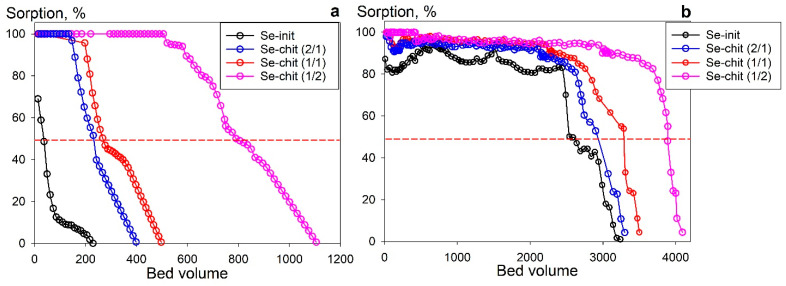
Sorption of uranium under dynamic conditions from model solutions 0.1 mol L^−1^ NaNO_3_, (**a**) pH 6, (**b**) pH 8.

An important feature of sorbents is their suitability for dynamic sorption conditions, which ensure the continuity of the sorption process. In view of this, a study of the possibility of using sorbents under dynamic sorption conditions was conducted. Figure 14 shows the sorption curves of uranium from model solutions with pH 6 (Figure 14a) and pH 8 (Figure 14b). It is shown that Se-chit (x/y) composite materials have the highest sorption resource, as evidenced by the values of the total dynamic sorption capacity (*TDEC*, up to 100% infiltration of uranium into the filtrate): 0.66 mmol g^−1^ (Se-chit (2/1)), 0.72 mmol g^−1^ (Se-chit (1/1)), and 0.95 mmol g^−1^ (Se-chit (1/2)) for sorption from a solution with pH 6, and 0.84 mmol g^−1^ (Se-chit (2/1)), 0.95 mmol g^−1^ (Se-chit (1/1)), and 1.50 mmol g^−1^ (Se-chit (1/2)) for sorption from a solution with pH 8. The values of total dynamic exchange capacity (*TDEC*) for Se-init were 2–8 times lower, which is likely due to the increased availability of adsorption centers in composite adsorbents, as well as the sorption of uranium throughout the entire grain volume.

Percolation of the model solution at a rate of 10 b.v./h resulted in effective cycles of filtration (50% uranium slip into the filtrate); the decrease in a row was as follows: Se-chit (1/2) > Se-chit (1/1) > Se-chit (2/1) > Se-init. The *TDEC* values were 50–800 b.v. for the solution with pH 6 and 2700–4000 b.v. for the solution with pH 8. These results indicate that the use of a natural biopolymer as a matrix contributes to the production of highly efficient sorption composite materials with increased kinetic and capacitive sorption characteristics. Further studies of sorption columns, with the establishment of the optimal percolation rate of model solutions of natural and anthropogenic origin, will allow us to determine the boundary operating conditions of the adsorbents.

## 3. Conclusions

Thus, a simple approach to the synthesis of new composite sorbent materials based on a natural chitosan polymer matrix and a Se derivative of 4-amino-N′-hydroxy-1,2,5-oxadiazole-3-carboximidamide was developed in the work. A series of physicochemical studies indicate that the formation of an amidoxime film on the matrix surface occurs due to strong covalent binding, while preserving organo-functional groups.

Under static sorption conditions, it was found that the materials exhibit enhanced sorption-selective properties toward the radionuclide. The presence of primary components, which facilitate the formation of multinuclear uranium complexes, had little effect on the sorption efficiency. High levels of uranium sorption persist within the pH range of 4–9: *K_d_* values were about 10^5^–10^6^ mL g^−1^.

The formation of the Se derivative of 4-amino-N′-hydroxy-1,2,5-oxadiazole-3-carboximidamide during the grafting of the polymer matrix (Se-chit) yields a chemically more stable and efficient adsorbent compared to the materials synthesized by the direct binding of 4-amino-N′-hydroxy-1,2,5-oxadiazole-3-carboximidamide with chitosan (43AF-chit). This enhanced effectiveness is attributed to the unique distribution of the sorption-active component throughout the grain volume of the material. However, excessive chitosan in the composite may lead to gradual dissolution and a transition to a gel-like state, potentially resulting in the formation of liquid radioactive waste with a complex chemical composition.

In comparison to non-composite adsorbents, the sorption capacity of uranium for the composites increases by 1.5 times, reaching approximately 450 mg g^−1^. Furthermore, the sorption kinetic parameters improve by 2 to 10 times for composites with amidoxime/chitosan ratios of 1:1 and 1:2. This enhancement can be attributed to the developed surface morphology and ion permeability of chitosan.

It was shown that under dynamic sorption conditions, the effective filter cycle values decrease in the following order: Se-chit (1/2) > Se-chit (1/1) > Se-chit (2/1) > Se-init, with approximate values of 50–800 b.v. for solutions with pH 6 and 2700–4000 b.v. for solutions with pH 8. These results indicate that the synthesized sorbents are promising materials for uranium removal from liquid media, suitable for both purification and the recovery of radionuclides as valuable raw materials.

## 4. Materials and Methods

Selenium dioxide, acetic acid, ethanol, and methylene chloride of the especially pure grade were used to prepare the sorption material (Nevareaktiv LLС, St. Petersburg, Russia). Chitosan was purchased from Nevareaktiv LLC (St. Petersburg, Russia); the degree of acetylation was 0.25; the viscosity–average molecular weight was 250 kDa. To prepare working model solutions, metal salts of the chemically pure grade were used without additional purification—these were purchased from Nevareaktiv LLC. The radionuclide U-238 in the form of UO_2_(NO_3_)_2_ of the especially pure grade was used as a sorption element (LLC GRANKHIM, Chelyabinsk, Russia).

### 4.1. Synthesis of the Se-Derivative 4-Amino-N′-hydroxy-1,2,5-oxadiazole-3-carboximidamide [11]

To obtain the non-composite material, 4-amino-N′-hydroxy-1,2,5-oxadiazole-3-carboximidamide was brought in contact with Se(IV) oxide in a 1:1 molar ratio. Acetic acid was added to the mixture, after which the resulting solution was boiled with intense stirring. Mixing was carried out using an electromagnetic stirrer equipped with a heating function, at a mixing speed of 400–600 rpm. After 80–100 min, a dark purple precipitate was formed. After the formation of the precipitate was complete, the mixture was cooled to room temperature, and the reaction product was separated from the mother liquor on a “blue ribbon” filter (pore size 2–3 microns). Unreacted components of the original mixture were removed. For this purpose, the resulting precipitate was washed sequentially with cold distilled water, ethyl alcohol and methylene chloride. The resulting product was dried to a constant weight for 24 h.

### 4.2. Synthesis of Composite Sorbents Based on Chitosan and Se-Derivative 4-Amino-N′-hydroxy-1,2,5-oxadiazole-3-carboximidamide

A mixture of chitosan with dioxane and acetic acid was added to a pre-dissolved (in acetic acid) mixture of 4-aminofurazane-3-carboxamidoxime and selenium dioxide (with a molar ratio of 1:1) during boiling. The obtained mixture was boiled in acetic acid with a reflux condenser for 80–100 min. At the end of the reaction, the mixture was cooled to room temperature. Subsequently, all components were transferred to methanol to separate the unreacted components of the initial mixture. The sediment was filtered using a “blue ribbon” filter and washed sequentially with ethyl alcohol and methylene chloride to remove impurities. The resulting product was dried to a constant weight in a vacuum desiccator at 5–10 mmHg above P_2_O_5_, at a temperature of 20–25 °C for 24 h. The final product was a dark orange powder with a brown tinge, with a grain size of 0.05–0.2 mm. By varying the ratio of the initial components of amidoxime/chitosan, five types of composite materials were obtained, labeled as Se-chit (2/1), Se-chit (1/1), Se-chit (1/2), Se-chit (1/4), and Se-chit (1/6).

### 4.3. Synthesis of Composite Sorbents Based on Chitosan and 4-Amino-N′-hydroxy-1,2,5-oxadiazole-3-carboximidamide

A chitosan suspension was added to a methanol solution of 4-amino-N′-hydroxy-1,2,5-oxadiazole-3-carboximidamide with active stirring. Mixing was continued until the reaction mixture was completely homogenized. Finally, the resulting mixture was filtered through a “blue ribbon” filter and washed with distilled water to separate the unreacted reaction products.

The resulting product was dried to a constant weight in a vacuum desiccator at 5–10 mmHg above P_2_O_5_, at a temperature of 20–25 °C for 24 h. The final product was a light-yellow powder, with a grain size of 0.05–0.2 mm. By varying the ratio of the initial components of amidoxime/chitosan (0.7/11.8 mol, 2.1/10.6 mol, 3.5/9.3 mol), three types of composites were synthesized, labeled as 43AF–chit (5), 43AF–chit (15), 43AF–chit (25).

### 4.4. Evaluation of Sorption Characteristics Under Static Conditions

In the sorption experiments, initially, the U(VI) salt (nitrate) was used. However, to simplify the description, hereinafter, U(VI) and its possible forms of existence will be marked as just uranium.

Study of sorption properties of the materials under static conditions was carried out by removal of uranium from solutions with a volume to mass ratio of 1000 mL g^−1^. Mixing was conducted for 24 h using an orbital shaker. The oscillation amplitude and rotation speed were 10 mm and 150 rpm, respectively. The experiment began with the sorbents being kept in a model solution without uranium for one day. After separating the solution from the sorbent, the model solution with uranium content ranging from 20 to 30 mg L^−1^ was added. After 24 h of stirring, the uranium-containing model solution was separated from the sorbent by filtration through an acetyl cellulose filter with a pore size of 45 µm. The residual uranium content in the filtrate was determined using Arsenazo III at a wavelength of 656 nm [46]. The results obtained were used to further calculate the extraction efficiency (*S*, %) and the uranium distribution coefficients (*K_d_*, mL g^−1^).

The extraction efficiency was calculated according to Equation (1) as follows:

(1) $$S=\left(1-\left(\frac{C_{e}}{C_{i}}\right)\right)\times 100$$

The value of the uranium distribution coefficient (mL g^−1^) was calculated using Equation (2):

(2) $$K_{d}=\frac{C_{i}-C_{e}}{C_{i}}\times \frac{V}{m}$$

where *С_i_*—the initial concentration of uranium in the model solution (mg L^−1^), *C_e_*—the equilibrium residual concentration of uranium in the solution after filtration (mg L^−1^), *V*—the volume of the solution (mL); *m*—the weight of the sorbent sample (g). Using the initial and equilibrium pH values of the solution, the zero charge point values (*pH_pzc_*) were found by a graphical method. The results are presented as a graphical dependence of the difference between the initial values of the pH of the solution and the equilibrium value of the pH of the solution (pH_init_ − pH_eq_) on the initial values of the pH of the solution (*pH_init_*). The intersection point of the abscissa axis and the ordinate axis with a value equal to zero corresponds to the value of pH_pzc_, which is the pH value of the solution at which the sorbent surface is not charged.

The influence of the presence of anions in the solution on the static exchange capacity of uranium sorption was evaluated using solutions of sodium salts (HCO_3_^−^, NO_3_^−^, SO_4_^2−^). For a deeper evaluation of the changes in the sorption properties of adsorbents under static conditions and in the presence of competing ions, the concentration of these ions in the solution was varied from 0.001 mol L^−1^ to 0.1 mol L^−1^.

At the end of the experiment, the equilibrium uranium content in the solution was estimated to calculate the values of static exchange capacity (SEC, mg g^−1^) according to the following equation:

(3) $$SEC=\frac{\left(C_{i}-C_{e}\right)\times V}{m}$$

where *С*_i_—the initial concentration of uranium in the model solution (mg L^−1^), *C_e_*—the equilibrium residual concentration of uranium in the solution after filtration (mg L^−1^), *m*—the weight of the sorbent sample (g), *V*—the volume of the solution (L).

Using uranium adsorption isotherms, the nature of the adsorption process was evaluated. For this purpose, a series of samples with the masses of adsorbents ranging from 0.001 to 2 g were brought into contact with model solutions containing 0.01 mol L^−1^ NaNO_3_, and 30 mg L^−1^ uranium. The acidity of the model solutions was varied between pH 6 and 8. The experimental process involved identical processing steps as in the previous experiments. The standard Langmuir (4), Freundlich (5) and SIPS (6) equations were used to describe the sorption isotherm:

(4) $$G=G_{max}\times \frac{K_{l}\times C_{e}}{1+K_{l}\times C_{e}}$$

(5) $$G=K_{f}\times C^{m}$$

(6) $$G=G_{max}\times \frac{K_{lf}\times C^{m}}{1+K_{lf}\times C^{m}}$$

where *G_max_*—the maximum sorption value (mg g^−1^), *C*—the uranium concentration in the solution (mmol L^−1^), *K_f_*—the Freundlich constant ((mmol/g) × (L/mmol)) describing the relative adsorption capacity and representing the value of adsorption at an equilibrium concentration equal to one, *K_l_, K_lf_*—the adsorption equilibrium constants that characterize the energy of the adsorbent–adsorbate bond (L/g), *m*—the indicator of heterogeneity of exchange sites that characterizes the change in the heat of adsorption depending on the degree of their filling.

The experimental data were approximated by means of the specified equations using the SciDAVis software (version 1.23).

The kinetic parameters of the uranium sorption process were evaluated using a model solution containing 0.1 mol L^−1^ sodium nitrate and 20–30 mg L^−1^ uranium in the form of uranyl nitrate. Two types of model solutions were used in this work, with pH 6 and pH 8. A 1 mol L^−1^ NaOH solution was used to adjust the pH of the solution. The ratio of liquid to solid phase was 200 mL g^−1^. After the adsorbent was brought into contact with the model solution, an aliquot of the solution was taken after a certain time interval to determine the residual amount of uranium in the solution. The total sorption time did not exceed 48 h.

To determine the limiting stage and estimate the kinetic parameters of the ion ex-change process, the Boyd–Adamson Equation (7) [47] was utilized. This equation allows for the calculation of the effective diffusion coefficient using the values of the diffusion rate constant, using the following equation:

(7) $$F=\frac{Q_{t}}{Q_{max}}=1-\frac{6}{\pi^{2}}\times e^{-Bt}$$

where *Q_t_*—the concentration of uranium [mg L^−1^] on the sorbent at time t; *Q_max_*—the concentration of uranium [mg L^−1^] on the sorbent at maximum sorption, *B*—the diffusion rate constant (s^–1^), which is described by Equation (8):

(8) $$B=\frac{\pi^{2}D_{i}}{r^{2}}$$

where *D_i_*—the effective diffusion coefficient [cm^2^ min^−1^], *r*—the radius of the ionite grain [cm].

The dependence of *Bt* as a mathematical function on *F* is described by Equation (9) and is given in the form of tabular values by Richenberg in [48].

(9) $$Bt=6.28318-3.2899F-6.28318({1-1.0470)}^{\frac{1}{2}},\;at\;F\leq 0.85$$

where *B_t_*—Fourier homochrony criteria.

The value of B was determined as the tangent of the angle of inclination of the straight line obtained by approximating the initial values of the kinetic curve (for F < 0.5) in the coordinates B_t_ = f(t). With the value of B known, Di was calculated using Equation (8).

### 4.5. Evaluation of Sorption Characteristics Under Dynamic Conditions

The study of the dynamic characteristics of sorption was conducted under continuous flow conditions using a model solution with a pH of 6 or 8, containing 0.1 mol L^−1^ NaNO_3_. The solution was passed through a fixed sorbent layer with a volume of 1 mL (H_layer_ = 4.8 cm) and a grain size of 0.05–0.2 mm at a rate of 10 b.v. h^−1^ in a glass column with an inner diameter of 0.6 cm and a wall thickness of 0.1 cm. Prior to this, the materials were soaked in a model solution that did not contain uranium.

Filtrates after the column were collected by fractions, and the uranium concentration was determined. The extraction efficiency was calculated according to Equation (1) in each sample separately.

*TDEC* was calculated by integrating the curves that represent the dependence of adsorption on the volume of solution passed, as shown in Equation (10):

(10) $$TDEC=\int_{0}^{n}f\left(\frac{\left(C_{0}-C_{i}\right)V_{i}}{m}\right)dV_{t}$$

where *С*_0_—the initial concentration of uranium in the model solution, [mmol mL^−1^]; *С_i_*—the concentration of uranium in the i eluate fraction, [mmol mL^−1^]; *V_i_*—the volume of the *i* eluate fraction, [ml]; *m*—sorbent weight, [g]; Vt—the total solution volume, [mL].

### 4.6. Equipment

To establish the peculiarities of the molecular structure formation of the samples, a combination of physicochemical methods was employed. The determination of the types of chemical bonds in the polymer network was performed by infrared spectroscopy using a Spectrum 1000 spectrometer manufactured by Perkin Elmer (Waltham, MA, USA) in KBr pellets.

To determine the phase composition of the obtained materials and to study the nature of the interaction between the matrix and the sorption-active component, X-ray diffraction patterns were recorded using a Colibri X-ray diffractometer manufactured by JSC IC Burevestnik (St. Petersburg, Russian Federation), with CuKα-radiation in the angle range of 2° < 2θ < 90° in the point-by-point scanning mode.

Investigations of the morphology of the surface of the obtained composites were performed using high-resolution scanning electron microscopy with a HITACHI TM 3000 device (HITACHI, Tokyo, Japan), at accelerating voltages of 5–15 kV and a beam current of I ≈ 100 pA. The device was equipped with an accessory for energy dispersion analysis (EDA) by Bruker (Waltham, MA, USA).

The specific surface area and pore size of the material were determined by a low-temperature nitrogen adsorption technique using an Autosorb IQ device by Quantachrome Instruments (New York, NY, USA). The calculation was performed according to the BET method (the theory of Brunauer–Emmett–Teller).

The control of the elemental composition of model samples for studies of sorption-selective properties of materials was performed using the atomic absorption flame spectroscopy using a Thermo Solar AA M6 device manufactured by Thermo Electron Corporation (Waltham, MA, USA). The content of uranyl ions in the solution was determined by the spectrophotometric method using a Shimadzu UV-1800 spectrophotometer (Tokyo, Japan) in the wavelength range from 600 to 800 nm. The calibration curve for uranium content had a radionuclide concentration range from 0.1 mg L^−1^ to 30 mg L^−1^.

## Figures and Tables

**Table 1:** Synthesis parameters and abbreviation name of sorption materials.

	Se-Init	43AF-Chit (5)	43AF-Chit (15)	43AF-Chit (25)	Se-Chit (2/1)	Se-Chit (1/1)	Se-Chit (1/2)	Se-Chit (1/4)	Se-Chit (1/6)
Mass ratio of Se-init/chitosan	100/0	-	-	-	65/35	50/50	35/65	20/80	15/85
Mass ratio of 43AF/chitosan	-	5/95	15/85	25/75	-	-	-	-	
Molar ratio of Se-init (43AF)/chitosan	-	0.7/11.8	2.1/10.6	3.5/9.3	2/1	1/1	1/2	1/4	1/6
Curing temperature	60 °C	60 °C, 120 °C, 160 °C							

**Table 2:** Constants of the Langmuir, Freundlich and SIPS equations calculated after approximating the experimental data.

Equation	Parameter	Se-Init	Se-Chit (2/1)	Se-Chit (1/1)	Se-Chit (1/2)
Langmuir	*G_max_*, mg g^−1^	380 ± 20	470 ± 15	480 ± 15	420 ± 15
	*K_l_*	0.24 ± 0.05	0.11 ± 0.03	0.12 ± 0.02	0.10 ± 0.02
	*R* ^2^	0.988	0.873	0.939	0.940
Freundlich	*K_f_*	100 ± 20	44 ± 15	45 ± 10	44 ± 9
	*m*	0.440 ± 0.080	0.682 ± 0.101	0.668 ± 0.047	0.622 ± 0.057
	*R* ^2^	0.820	0.924	0.982	0.981
SIPS	*G_max_*, mg g^−1^	370 ± 15	470 ± 15	480 ± 15	420 ± 15
	*K_lf_*	0.304 ± 0.029	0.022 ± 0.009	0.0453 ± 0.012	0.038 ± 0.003
	*n*	1.694 ± 0.114	1.936 ± 0.226	1.542 ± 0.133	1.221 ± 0.046
	*R* ^2^	0.932	0.985	0.98	0.999

**Table 3:** Kinetic and sorption-selective parameters of uranium adsorption from model solutions with pH 6 and pH 8.

No.	Parameters	pH 6				pH 8			
		Se-Init	Se-Chit (2/1)	Se-Chit (1/1)	Se-Chit (1/2)	Se-Init	Se-Chit (2/1)	Se-Chit (1/1)	Se-Chit (1/2)
1	*t_max_* × 10^−2^ min	28.8	14.4	14.4	14.4	14.4	14.4	2.4	2.4
2	*K_d_* × 10^−3^, ml g^−1^	3.0	9.3	7.6	1433.0	7.0	49.0	70.0	960.0
3	*D_i_* × 10^7^, cm^2^ min^−1^	5.7	6.3	20.5	40.5	30.8	16.5	38.2	42.2
4	*T*_1/2_, min	300	270	80	40	75	80	45	40
5	** *R*^2^	0.975	0.972	0.978	0.996	0.989	0.94	0.990	0.984

** *R*^2^—The value of the correlation coefficient of linear dependence *Bt* = *f*(*τ*).

## Data Availability

The original contributions presented in this study are included in the article. Further inquiries can be directed to the corresponding authors.
